# Time Motion Analysis of Emergency Physician Workload in Urgent Care Settings

**DOI:** 10.5811/westjem.41536

**Published:** 2025-07-09

**Authors:** Scott Odorizzi, Jessica Hogan, Sabrain Idris, Loraina Marzano, Veronique Rowley, Max Yan, Yuxin Zhang, Jeffrey J. Perry

**Affiliations:** * University of Ottawa, Department of Emergency Medicine, Ottawa, Ontario, Canada; † The Ottawa Hospital Research Institute, The Ottawa Hospital, Ottawa, Ontario, Canada

## Abstract

**Introduction:**

The Predictors of Workload in the Emergency Room (POWER) study, published in 2009 using data from 2003, examined the workload of emergency physicians using the Canadian Triage and Acuity Scale (CTAS) as a surrogate marker. Many hospitals use a case-mix formula incorporating annual census and POWER’s study data to determine staffing levels. However, significant changes in emergency medicine have occurred since its publication, including the implementation of electronic health record systems, increased patient complexity, real-time dictation software, and human health resource challenges due to the COVID-19 pandemic. In this study we aimed to quantify the time required to perform tasks during the care of ambulatory emergency department (ED) patients. Our secondary objective was to stratify these times based on CTAS and clinician factors.

**Methods:**

We conducted a prospective observational time-motion study in the urgent care section of a tertiary-care, academic ED with 90,000 visits annually, 70% of which are ambulatory. Research assistants shadowed physicians on two 8-hour shifts daily (8 am-12 am) from July 12–August 14, 2022, tracking the time taken by physicians to perform tasks. We calculated aggregate task times per patient.

**Results:**

We observed 1,204 patient encounters over 65 shifts by 37 unique physicians. The mean treatment time was 21.6 minutes (95% confidence interval [CI] 19.9 – 23.3) for ambulatory CTAS 2 patients; 22.5 minutes (95% CI 21.2 – 23.6) for CTAS 3 patients; 19.7 minutes (95% CI 17.9 – 21.6) for CTAS 4 patients; and 17.4 minutes (95% CI 14.9 – 19.9) for CTAS 5 patients. Compared to the previous 2003 POWER study data, CTAS 4 and 5 patient assessment times took 31% and 58% longer, respectively. Total assessment time by CTAS was statistically significant only comparing CTAS 5 patients to all others (*P* = .02). Physicians who dictated their charts spent 34% less time (2.1 minutes per patient) charting than those who typed them.

**Conclusion:**

The average time to see an ambulatory ED patient was 21.7 minutes. Low-acuity urgent care patients take longer to assess now than 20 years ago. The CTAS alone is a poor marker of workload for ambulatory patients, necessitating a reassessment of staffing and compensation formulas.

## INTRODUCTION

Emergency departments (ED) in Canada are facing unprecedented levels of strain and crowding.^1^ Emergency departments must find ways to become increasingly efficient with fewer relative resources to meet the demands they face. As patient wait times increase, ensuring appropriate physician staffing of the ED is critical. Currently, physician staffing in many Ontario hospitals is based on a case-mix formula that combines ED census and acuity^2^; the Canadian Triage and Acuity Scale (CTAS) is used to determine acuity. The CTAS is a five-level system with level 1 representing the most acute patients and level 5 representing non-urgent patients.^3^ This formula allows a set number of minutes of emergency physician time for each patient based on their CTAS score. This ultimately translates into the number of annual hours of coverage that physicians are paid to provide.

The Predictors of Workload in the Emergency Room (POWER) study,^4^ published in 2009 using data from 2003, attempted to understand emergency physicians’ workload for the first time in Canada. The study tracked physicians over their entire shift and timed various activities related to patient care, ultimately describing the time spent on various tasks and the total time spent assessing and treating all ED patients by CTAS level. While this was the first study in Canada to provide a data-driven emergency physician workload by CTAS, the authors questioned the accuracy of CTAS alone as a predictor of workload.

Previous studies have demonstrated significant changes to the practice of emergency medicine since the POWER study’s publication in 2009, including delays in care due to medical complexity, 5 the use of electronic health records (EHR), 6,7 the widespread adoption of real-time dictation software for documentation,^8^ and the effects of the COVID-19 pandemic on human healthcare resources.^9^ However, the overall difference in time spent to manage low-acuity patients has not been robustly assessed since the original POWER study. Further, the POWER study considered ED patient factors affecting physician workload but did not collect data on the physicians themselves. There has since been research that questions physician gender differences in assessment^10^ and documentation times^11^ in ambulatory clinics. Hypothetically, there may also be differences in care times based on physician seniority.

Our study objective was to quantify the time required to perform tasks in the care of ambulatory ED patients. Our secondary objective was to stratify these times based on assigned CTAS and by clinician factors.

## METHODS

### Study Design and Time Period

We conducted a prospective, observational, time-motion study to track the amount of time taken by physicians to perform tasks in the care of patients in the urgent care area of a tertiary-care, academic ED with 90,000 patient visits per year. This study took place from July 12–August 14, 2022. This study received research ethics board exemption.

**Population Health Research Capsule**

What do we already know about this issue?

*Emergency department workload is often estimated using outdated triage-based models and informs staffing models leading to understaffed EDs*.

What was the research question?

*How much time do physicians spend on low-acuity ambulatory ED patients, and how does this vary by acuity and clinician factors?*

What was the major finding of the study?

*The mean treatment time was 19.7 minutes (95% CI 17.9 – 21.6) for Canadian Triage and Acuity Scale (CTAS) 4 patients, and 17.4 minutes (95% CI 14.9 – 19.9) for CTAS 5 patients*.

How does this improve population health?

*Ambulatory, low-acuity ED patients now take significantly longer to treat. It is time to reassess ED workload-based compensation and staffing models*.

### Study Setting and Population

This study took place at The Ottawa Hospital, General Campus ED, in the urgent care area. The urgent care area sees low-acuity ambulatory patients not requiring heavy nursing resources or cardiac monitoring. It has 24 assessment spaces open 24 hours/day with up to quadruple (50–64 hours/day) physician coverage. There are 3–4 nurses in the area depending on time of day, and the unit sees ~160 patients per day, 65–70% of our entire ED daily volume. The emergency physician group at The Ottawa Hospital consists of approximately 90 physicians and provides physician services between both campuses. Both EDs are part of tertiary-care, academic teaching hospitals.

The Ottawa Hospital, General Campus, is a teaching hospital. Most shifts have 1–2 learners assigned to them including medical students and residents from all postgraduate training programs. The hospital uses Epic’s EHR (Epic Systems Corporation, Verona, WI), and Dragon dictation software (Microsoft Dragon, Microsoft Corporation, Redmond, WA) is available for staff use. No scribes are employed in the ED.

### Intervention

Our study protocol was adapted from the previous POWER study.^4^ Research assistants (RA) were assigned to follow staff physicians on two shifts each day over the study period, from 8 am–12 am (ie, 16 hours per day). The first shift ran from 8 am–4 pm and the second shift from 4 pm–12 am. At the beginning of the shift, the RAs would collect information on physician age, sex, years in practice, years working at The Ottawa Hospital, their training stream (Canadian College of Family Physicians vs Royal College of Physicians and Surgeons of Canada), and charting type (dictating, typing, or mix). Following this, they would shadow staff physicians without interacting with them for the duration of the shift while timing the tasks of interest. The RAs did not enter patient rooms and had no direct contact with patients. Neither did they directly shadow postgraduate medical trainees, medical students, or nurse practitioners assigned to work with the staff physician. Night shifts were not tracked as there was no RA shift covering the urgent care area, which started at midnight, precluding the entire shift from being observed.

Before the initiation of the study, an email was sent to all physicians explaining the purpose of the study and gave the option to not participate. Only one physician chose not to participate; however, this physician was not scheduled to work the study shifts during the study period.

### Outcome Measures

We designed a custom application to operate on a handheld Galaxy S8 tablet (Samsung Electronic Corporation, Ltd, Suwon, South Korea). The application allowed RAs to input patient information (age, CTAS score, and presenting complaint) and record the time spent on tasks attributable to that patient. The application allowed for a dynamic list of patients under the care of the observed physician. Patients were added as they were signed up for and removed from the list after they were physically discharged from the ED and their chart had been fully completed by the physician. Patients with incomplete data by the end of the RA’s shift were right censored.

The primary outcome measures were task times per patient in eight pre-defined tasks, in seconds. These tasks were as follows: 1) initial assessment—time the staff emergency physician spent on initial assessment of the patient; 2) re-assessment—time spent with the patient after an initial assessment had already taken place; 3) discussion with learners—time spent reviewing learners’ cases and teaching, outside the patient room; 4) seeing learners’ patients—time spent in the room assessing the learner’s patient; 5) charting—any time spent documenting, billing, or entering orders; 6) reviewing information on the computer—any time spent reviewing past documentation, triage notes, medications, and past medical history; 7) phone—any time spent on the phone in consultation with specialty services (obtaining collateral history on the phone was captured under initial assessment); and 8) not related to patient care—any time spent that was not related to patient care such as down time, eating, attending the restroom, socializing, etc.

The raw data included the summation of time, in seconds, spent on each task per patient with the associated patient and emergency physician characteristics.

### Data Analysis

Task times were tracked in seconds and analyzed with descriptive statistics. We conducted Wilcoxon rank-sum tests for comparing task times with non-parametric distributions and *t*-tests to compare task times with normal distributions. Linear regression analysis was conducted to compare task times and total patient assessment times by CTAS and for physician characteristics. We performed all analyses using SAS 9.4 (SAS Institute Inc., Cary, NC).

## RESULTS

We gathered data on 1,204 unique patient encounters over 65 shifts by 37 unique physicians. Characteristics of the study physicians are presented in Table 1. Twelve (32.4%) study physicians were women. Five (13.5%) physicians typed their charts as opposed to dictating or using a mix of typing and dictating. Twenty-five (67.6%) of the physicians were trained in emergency medicine in a Royal College residency program with the remaining 12 (32.4%) trained by the College of Family Physicians. Twelve (32.4%) of the physicians had been practicing for < five years. The amount of physician time spent on an individual task is shown in Table 2. On average, physicians spent 93.5% of their time on shift related to patient care activities. The mean assessment time of any patient was 21.7 minutes (95% CI [confidence interval] 20.6 – 22.2). When performing an initial assessment of a patient themselves, staff physicians spent 7.4 minutes doing so. As a teaching hospital, a significant amount of time is spent on teaching and reviewing learners’ patients (5.9 minutes) and then seeing their patients (7.6 minutes), when this occurred; 32% of patients were initially seen by learners, and 62% of patients seen by a learner were also seen by a staff physician. For patients seen by a staff physician only, the mean total assessment time was 27.8 (SD 14.6) minutes. For patients seen by learners, the mean total staff physician time overseeing this was 15.6 (SD 13.7) minutes. Of all patients observed, 0% were categorized as CTAS 1; 20.6% as CTAS 2; 52.4% as CTAS 3; 17.4% as CTAS 4; and 7.3% as CTAS 5 Table 3 presents the data from individual tasks and total assessment times by CTAS. The mean treatment time was 21.6 minutes (95% CI 19.9 – 23.3) for ambulatory CTAS 2 patients; 22.5 minutes (95% CI 21.2 – 23.6) for CTAS 3 patients; 19.7 minutes (95% CI 17.9 – 21.6) for CTAS 4 patients; and 17.4 minutes (95% CI 14.9 – 19.9) for CTAS 5 patients. Total assessment time by CTAS score was only statistically significant when comparing CTAS 5 patients to all others (*P* = 0.022). There was significant variability of assessment times within each CTAS category. However, the sample size of our study resulted in narrow 95% CIs for the point estimates.

There was a significant difference by sex in the overall time spent on any given patient, with female physicians spending 23.3 minutes and males spending 21.0 minutes (+10.9%, *P* = .03). There was no significant difference in any individual task measured, with the exception of charting in which women spent on average 7.4 minutes per patient compared to men who spent 6.3 minutes (+17.7%, *P* = .002). Physicians who dictated their charts spent the least amount of time charting (6.1 minutes) per patient compared to a mix of typing/dictating (6.3 minutes, *P* = .54), or typing alone (8.2 minutes, *P* < .001). We found no differences in task times when comparing physician seniority (≤ 5 years vs > 5 years as staff) or training stream (RCPSC vs CCFP-EM).

## DISCUSSION

### Interpretation of Findings

Our study showed that the CTAS is not a good indicator of expected workload times for emergency physicians when studying ambulatory patients, with only CTAS 5 patients having a statistically significantly lower workload demand and ambulatory CTAS 2–5 patients being nearly equal in their workload demands. This is the first study to our knowledge that has examined clinician factors associated with the workload demands of attending emergency physicians. One previous study assessed fully licenced attending physicians vs trainees and found that trainees spent significantly more time with patients.^12^ In this, study we focused only on attending physicians.

We found no difference in physician training stream or seniority in the time spent on any task or total time caring for patients. We did find differences based on clinician charting method: Those who typed their charts spent 34% longer charting than those who dictated. Interestingly, we did find differences by sex in our study, but these were limited to charting time and total assessment time. The differences in charting time between men and women did not account for the full difference in total assessment time, suggesting other factors or tasks that differed; however, we did not examine those factors. This may include time talking to families after initial patient assessment or interruptions by staff, patients, or families while walking to and from patient assessment rooms.

### Comparison to Previous Studies

The POWER study captured the workload of emergency physicians per patient, by CTAS level. The dataset from this study is now 20 years old; yet the same case-mix formula to calculate emergency physician staffing hours remains. Our study, which captured an updated set of data, showed significant differences in the workload of emergency physicians now compared to 2003. While the POWER study examined all patients in the ED and our study examined ambulatory ED patients, it is a fair assumption that almost all the CTAS 4 and 5 patients captured in the POWER study were triaged to ambulatory areas of an ED. The POWER study showed that physicians took 15 minutes and 11 minutes to care for these patients, respectively. Our study showed that these times have increased and now take 19.7 minutes and 17.4 minutes, respectively. This represents a 31% increase (4.7 minutes) in workload for CTAS 4 patients and 58% increase (6.4 minutes) for CTAS 5 patients. We hypothesize that the increase in workload reflects increasing patient complexity, workload increases associated with use of EHRs, and ED crowding.

With respect to CTAS 2 and 3 patients, it is more difficult to generalize these to the POWER study. A significant portion of CTAS 2 and 3 patients are triaged to non-ambulatory care areas of an ED, which we did not study. It is expected that higher acuity, non-ambulatory patients would take longer to assess as these patients are often sicker, undergo more studies, and have higher admission rates. This was reflected in the POWER study with all CTAS 2 patients taking 39 minutes to care for and all CTAS 3 patients taking 26 minutes, while we showed ambulatory CTAS 2 patients to require 21.6 minutes and ambulatory CTAS 3 patients to require 22.4 minutes to care for.

### Strengths and Limitations

This study was conducted in a single Canadian, tertiary-care academic ED, which may limit its generalizability to other EDs; however, triage systems in other countries have been shown to be valid at identifying low-acuity patients^13^ and similar changes have occurred in the practice of emergency medicine throughout the United States and elsewhere over the last 20 years. Further, many triage systems have been shown to be valid at identifying low-acuity patients. Our study was also brief, with observations occurring over a one-month period, which may not have reflected the seasonality that occurs with EDs. Finally, we examined patients in the urgent care area of our ED only, which limits its comparison to the POWER study, which examined all ED patients.

### Clinical Implications

This study provides an updated workload analysis of low-acuity, ambulatory ED patients in a tertiary- care, academic ED. It highlights increases in workload times in comparison to previous studies, which ultimately should translate into increased physician staffing to better match ED demand. Finally, physician characteristics impact assessment times: those who type their charts could consider dictating to improve efficiency.

### Research Implications

To our knowledge, this study was the first to look at attending physician characteristics in determining workload variables in emergency physicians. We found significant differences in charting times between those who dictate and those who type. Further studies could examine the workload impact of scribes compared to those who dictate their charts. iDfferences by sex were observed in charting and total assessment times in our study. The differences in charting times between men and women did not account for the entirety of the difference in total assessment times, indicating an untracked variable making up some of this difference. Future studies could be conducted to further examine this difference. Low-acuity patients take significantly longer to assess and manage than they did 20 years ago during the time of the POWER study; future studies could examine the factors that have contributed to the longer treatment times.

## CONCLUSION

The mean time to care for a patient in the urgent care area was 21.7 minutes, ranging from 17.4 minutes for CTAS 5 patients to 22.5 minutes for CTAS 3 patients. Compared to the POWER study conducted 20 years ago, ambulatory, low-acuity ED patients now take significantly more time to treat. Given we now have electronic health records, physicians should be encouraged to dictate notes. Finally, these findings suggest a need for reassessment of workload-based compensation models in ED settings due to the increased amount of time it takes to care for patients now than 20 years ago.

## Figures and Tables

**Table 1:** Characteristics of the 37 unique physicians observed by research assistants during the study period.

Physician characteristic	
Sex	
Male	25/37, 67.6%
Female	12/37, 32.4%
Years practicing emergency medicine	
Median (IQR)	9 (4 −23)
Years working at The Ottawa Hospital	
≤5	12/37, 32.4%
>5	25/37, 67.6%
Training Stream	
RCPSC	25/37, 67.6%
CCFP-EM	12/37, 32.4%
Charting Method	
Dictate	14/37, 37.8%
Type	5/37, 13.5%
Mix	18/37, 48.6%

*IQR*, interquartile range; *RCPSC*, Royal College of Physician and Surgeons of Canada; *CCFP-EM*, Canadian College of Family Physicians Certification in Emergency Medicine.

**Table 2:** Amount of emergency physician time spent on individual tasks, in minutes.

Task (n)	Time to Complete Task in Minutes Mean (SD)
Initial assessment n = 601	7.4 (4.4)
Re-assessment n = 496	4.8 (4.6)
Discussion with learners n = 797	5.9 (5.3)
Seeing learner’s patient n = 259	7.6 (6.7)
Charting n = 1,026	6.7 (5.2)
Reviewing info on computer n = 939	3.2 (3.7)
On phone n = 277	3.7 (3.3)

* Not all tasks were performed for each patient observed.

**Table 3:** Univariate linear regression of individual task durations by Canadian Triage and Acuity Scale, in minutes.

Task		Estimate (min)	Standard error	P-value
Initial assessment	Reference (CTAS 2)	7.6	0.41	
	CTAS 3	−0.1	0.48	.82
	CTAS 4	−0.3	0.59	.67
	CTAS 5	−1.1	0.81	.21
Re-assessment	Reference (CTAS 2)	5.6	0.48	
	CTAS 3	−0.7	0.55	.19
	CTAS 4	−1.1	0.70	.13
	CTAS 5	−2.0	0.92	.03
Discussion with learners	Reference (CTAS 2)	6.2	0.40	
	CTAS 3	−0.1	0.47	.77
	CTAS 4	−1.1	0.58	.06
	CTAS 5	−0.8	0.78	.30
Seeing learner’s patient	Reference (CTAS 2)	8.2	0.79	
	CTAS 3	−0.5	0.94	.60
	CTAS 4	−1.8	1.24	.16
	CTAS 5	−3.0	1.47	.04
Charting	Reference (CTAS 2)			
	6.6	0.35		
	CTAS 3	0.0	0.41	.99
	CTAS 4	0.3	0.52	.56
	CTAS 5	−0.5	0.69	.47
Reviewing info on computer	Reference (CTAS 2)	2.9	0.26	
	CTAS 3	0.6	0.30	.06
	CTAS 4	−0.5	0.38	.23
	CTAS 5	−0.3	0.54	.59
On phone	Reference (CTAS 2)	3.3	0.38	
	CTAS 3	0.1	0.46	.76
	CTAS 4	0.6	0.65	.36
	CTAS 5	1.3	0.97	0.18
Total assessment time	Reference (CTAS 2)	21.6	0.93	
	CTAS 3	0.9	1.10	.44
	CTAS 4	−1.9	1.38	.18
	CTAS 5	−4.2	1.82	.02

*CTAS*, Canadian Triage and Acuity Scale.
